# Characterizing Normal Upper Extremity Lymphatic Flow with ^99m^Tc In-House Dextran: A Retrospective Study

**DOI:** 10.3390/diagnostics14171960

**Published:** 2024-09-05

**Authors:** Wiroj Katiyarangsan, Putthiporn Charoenphun, Krisanat Chuamsaamarkkee, Suchawadee Musikarat, Kidakorn Kiranantawat, Chaninart Sakulpisuti, Kanungnij Thamnirat, Arpakorn Kositwattanarerk, Chanika Sritara, Wichana Chamroonrat

**Affiliations:** 1 Division of Nuclear Medicine, Department of Diagnostic and Therapeutic Radiology, Faculty of Medicine Ramathibodi Hospital, Mahidol University, Bangkok 10400, Thailand; ken_id442@hotmail.com (W.K.); ps4436@hotmail.com (P.C.); krisanat.chu@mahidol.ac.th (K.C.); soy_suchawadee@hotmail.com (S.M.); chaninart.sak@mahidol.ac.th (C.S.); kanungnij.thm@mahidol.edu (K.T.); arpakorn.kos@mahidol.edu (A.K.); chanika.sri@mahidol.edu (C.S.); 2 Division of Nuclear Medicine, Department of Radiology, Maha Vajiralongkorn Thanyaburi Hospital, Pathum Thani 12110, Thailand; 3 Master of Science Program in Medical Physics, Faculty of Medicine Ramathibodi Hospital, Mahidol University, Bangkok 10400, Thailand; 4 Division of Plastic and Maxillofacial Surgery, Department Surgery, Faculty of Medicine Ramathibodi Hospital, Mahidol University, Bangkok 10400, Thailand; kidakorn.plasticsurgery@gmail.com

**Keywords:** upper extremity, dextran, lymphoscintigraphy

## Abstract

Lymphoscintigraphy evaluates the lymphatic system using radiocolloid compounds like ^99m^Tc-sulfur colloid and ^99m^Tc-nanocolloid, which vary in particle size and distribution timing. A local in-house Dextran kit (15–40 nm) was developed in 2005 and began clinical use in 2008 to localize sentinel lymph nodes; diagnose lymphedema; and detect lymphatic leakage. The normal drainage pattern remains unexplored. We retrospectively analyzed 84 upper extremity lymphoscintigraphies from 2008 to 2021. ^99m^Tc in-house Dextran was intradermally injected into both hands, followed by whole-body imaging at specified intervals (≤15 min; 16–30 min; 31–45 min; 46–60 min), with some receiving delayed imaging. Visual and quantitative analyses recorded axillary and forearm lymph nodes and liver, kidney, and urinary bladder activity. Results showed 92% (77/84) upper extremity lymphatic tract visualization within 45 min. Axillary node detection rates increased from 46% (≤15 min) to 86% (46–60 min). Delayed imaging further revealed nodes. Epitrochlear or brachial node visualization was rare (4%, 3/84). Hepatic, renal, and urinary bladder activity was noted in 54%, 71%, and 93% at 1 h, respectively. The axillary node uptake ratio was minimal (<2.5% of injection site activity; median 0.33%). This study characterizes normal upper extremity lymphatic drainage using ^99m^Tc in-house Dextran, offering insights into its clinical application

## 1. Introduction

Lymphoscintigraphy, a low-risk nuclear imaging technique [1], is pivotal in diagnosing arm and leg lymphedema, initially described in 1953 [2]. It remains the gold standard for this diagnosis [3]. However, standardized procedures for upper extremity lymphoscintigraphy vary widely among diagnostic units due to factors like radiotracer type, injection site, and imaging methods [4,5,6,7,8,9].

Radiotracers in lymphoscintigraphy are typically colloid-based compounds usually labeled with technetium-99m pertechnetate, ideally under 100 nanometers for efficient lymphatic transport. Their size affects absorption and distribution speed during imaging sessions [10,11].

Reported radiotracers and their respective particle sizes include ^99m^Tc-human serum albumin (HSA), <4 nm; ^99m^Tc-dextran, <4 nm; ^198^Au-colloid, 5–15 nm; ^99m^Tc-microaggregated albumin, 10 nm; ^99m^Tc-antimony trisulphide (Lymph-Flo^®^), 3–30 nm; ^99m^Tc-albumin nanocolloid (Nanocoll^®^), <80 nm; ^99m^Tc-rhenium colloid (Nanocis^®^), 50–200 nm; ^99m^Tc-filtered sulfur colloid, <100 nm; ^99m^Tc-stannous fluoride (Hepatate^®^), <200 nm; ^99m^Tc-sulfur colloid (Cis-sulfur colloid^®^), 100–1000 nm; and ^99m^Tc-stannous phytate, 100–1000 nm [12,13,14,15,16,17,18,19,20,21,22,23,24,25,26,27].

Due to cost considerations and occasional shortages of imported colloid kits, local production of in-house colloid kits commenced in 2005. Derived from Dextran for its safety and availability, the kit features colloidal particles between 15 and 40 nanometers. This in-house Dextran kit was granted a Petty Patent by the National Department of Intellectual Property in October 2008. It has been optimized and preliminarily validated, demonstrating comparable efficacy in detecting sentinel lymph nodes compared to other nanocolloids. It is economically advantageous per kit or patient and particularly beneficial when testing multiple patients in a single day using a single bottle. Additionally, the kits can be stored for over a year and require straightforward preparation without boiling during labeling with ^99m^Tc [28].

In cooperation with the national public organization under the supervision of the Ministry of Higher Education, Science, Research and Innovation, this kit was recently commercialized in lyophilized form for nationwide distribution. Despite its current use in lymphoscintigraphy imaging, there remains a need for comprehensive data on the normal lymphatic drainage characteristics of ^99m^Tc in-house Dextran. We aim to characterize normal lymphatic drainage patterns using lymphoscintigraphy images with ^99m^Tc in-house Dextran from the upper extremities.

## 2. Materials and Methods

### 2.1. Study Population

This retrospective analysis includes all patients who underwent upper extremity lymphoscintigraphy at our facility from the introduction of the ^99m^Tc in-house Dextran in 2008 to 2021. Data collection included relevant demographic and clinical characteristics with inclusion and exclusion criteria as follows:

Inclusion Criteria:

- Aged 18 years and older.
- Patients with at least one normal arm.
- No previous diagnosis of lymphedema in the included arm.

Exclusion Criteria:

- History of arm or breast surgery on the included side.
- Prior radiotherapy to the included arm.
- Presence of cellulitis during examination.
- History of significant trauma to the included arm.
- History of cancer metastasizing to the included arm.
- Generalized edema.
- Peripheral vascular disease.
- History of bilateral breast cancer.
- Family history of congenital lymphedema.

### 2.2. Radiopharmaceutical Preparation and Administration

In total, 0.5 milliliters of the frozen in-house Dextran kit was thawed from −20 degrees Celsius before adding 0.5 milliliters of technetium-99m pertechnetate, approximately 185 MBq (5 mCi). The vial is gently agitated and left at room temperature for approximately 10 min. Quality control for labeling is performed using instant thin-layer chromatography impregnated with a silica gel (ITLC-SG), using methyl ethyl ketone (MEK) as the mobile phase to ensure radiochemical purity (RCP) exceeding 95% [28].

^99m^Tc in-house Dextran, 18.5 MBq (0.5 mCi), 0.2 mL, is injected intradermally into each hand dorsum. The patient is instructed to move their hands to facilitate radiopharmaceutical movement repeatedly.

### 2.3. Lymphoscintigraphy Imaging Procedure

**Figure 1:**
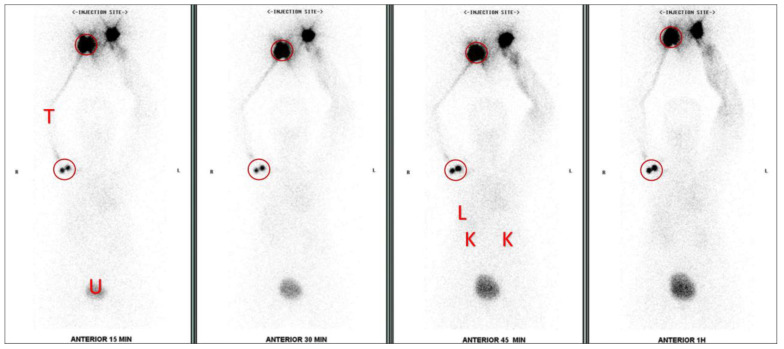
Anterior images at four time-points during the upper extremity lymphoscintigraphy protocol, illustrating sampled regions of interest (ROIs) indicated by circles. The ROIs were positioned over the normal upper extremity injection site (right hand) and the ipsilateral axillary lymph node in the right upper extremity. Notably, both hands exhibit injection site activity. However, no forearm lymph nodes were detected throughout the study. The 15-min image initially displays a lymphatic draining tract (T) in the right upper extremity and two right axillary lymph nodes, accompanied by fainter activity in the liver (L), kidney (K), and urinary bladder (U). In addition, lymphedema scintigraphy findings of the left upper extremity are evidenced by chronic dermal backflow and absence of typical lymphatic tract and node visualization, attributable to previous resection of left breast cancer.

Imaging is performed using a dual-head gamma camera (Philips ForteTM, Cleveland, OH, USA & GE InfiniaTM, Tirat Carmel, Israel), set in a fixed position with an H-mode start position for anterior and posterior views. The scan speed is 10 cm/min with a matrix size 256 × 1024. An LEGP collimator is used with a window centered at 140 keV ± 10% energy. The patient lies flat with their arm raised beside the head. Images are taken immediately after injecting the radiopharmaceutical in a continuous scan from the hands down to the pubic symphysis for 4 time-point intervals after radiopharmaceutical injection (≤15 min, 16–30 min, 31–45 min, and 46–60 min). Delayed imaging of up to 4 h may be performed. Figure 1 demonstrates 4-time-point images from the upper extremity lymphoscintigraphy protocol.

### 2.4. Qualitative (Visual) Analysis

Visualization of findings on lymphoscintigraphy images at each time-point includes the following:

- Axillary lymph nodes;
- Forearm (Epitrochlear of Brachial) lymph nodes;
- Upper extremity lymphatic tract and associated organs, i.e., liver, kidney, urinary bladder.

### 2.5. Quantitative Analysis

All available raw images were transferred and analyzed using Xeleris software (version 4.1; GE Healthcare, Bangkok, Thailand). The quantitative analysis involved assessing counts by drawing fixed-size regions of interest (ROIs) around the axillary node and the injection site on the ipsilateral normal hand as sampled ROIs in Figure 1. Two percentages were assessed using the following equation:

#### 2.5.1. Percentage of Axillary Lymph Node Uptake (%ANU) [29]


(1) $$\%\mathrm{ANU}=\frac{\mathrm{ANU}}{\mathrm{IIS}\times \mathrm{DF}}\times 100$$

ANU: Uptake of axillary node (counts)
IIS: Initial uptake in the injection site (counts)
DF: Technetium-99m’s decay factor

#### 2.5.2. Percentage of Radiopharmaceutical Clearance at Injection Site (%RC) [29]


(2) $$\%\mathrm{RC}=\frac{\left(\mathrm{IIS}\times \mathrm{DF}\right)-\mathrm{LIS}}{\mathrm{IIS}\times \mathrm{DF}}\times 100$$

IIS: Initial uptake in the injection site (counts)
LIS: Later uptake in the injection site at imaged time-points up to 1 h (counts)
DF: Technetium-99m’s decay factor at imaged time-points up to 1 h

### 2.6. Statistical Analysis

Continuous data are presented as mean ± standard deviation (SD) for normally distributed data and as medians and interquartile ranges for non-normally distributed data. Categorical data are presented as counts and percentages.

## 3. Results

### 3.1. Population Studied

**Figure 2:**
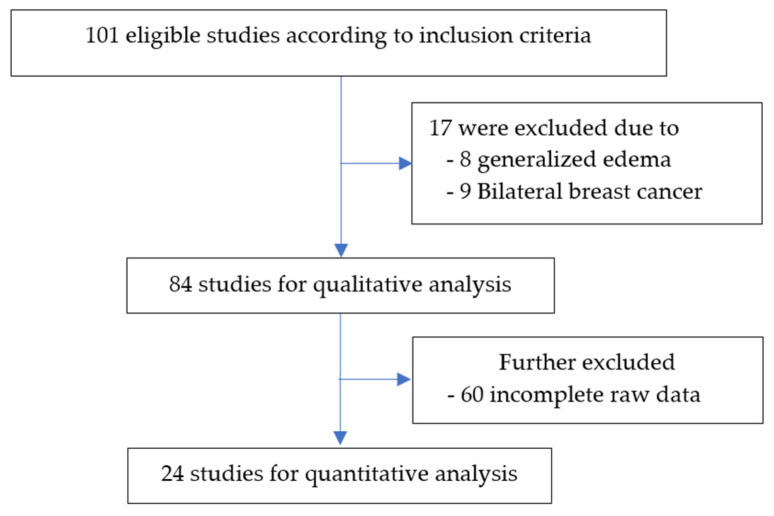
The sequence of participant selection criteria for inclusion and exclusion.

A total of 101 patients underwent lymphoscintigraphy studies using ^99m^Tc in-house Dextran between 2008 and 2021, meeting the study’s inclusion criteria. Seventeen patients were subsequently excluded: eight due to generalized edema and nine due to bilateral breast cancer. This exclusion resulted in 84 examinations qualifying for qualitative analysis. Notably, incomplete raw data in 60 studies limited the quantitative analysis to 24 examinations, as depicted in Figure 2.

### 3.2. Population Characteristics

Table 1 summarizes the characteristics of 84 patients eligible for qualitative analysis. The study population was predominantly female (96%) with a high prevalence of unilateral breast cancer history (92%). Among the remaining seven patients with unilateral arm swelling, causes included five cases of unknown origin, one related to Rhabdomyosarcoma, and one due to an accident. Both left and right arms were equally studied for visual analysis. The average age was 59.96 ± 12.15 years, with the cohort’s average weight, height, and BMI recorded as 64.36 ± 12.20 kg, 154.26 ± 6.00 cm, and 26.92 ± 4.64 kg/m^2^, respectively. All of the 24 patients included in the quantitative lymphoscintigraphy analysis were female. Nearly all, except one patient with unknown arm swelling, had a history of breast cancer. The average and standard deviation of age, weight, height, BMI, and affected side of the arm were comparable to those of the qualitative analysis group.

### 3.3. Qualitative Analysis

**Figure 3:**
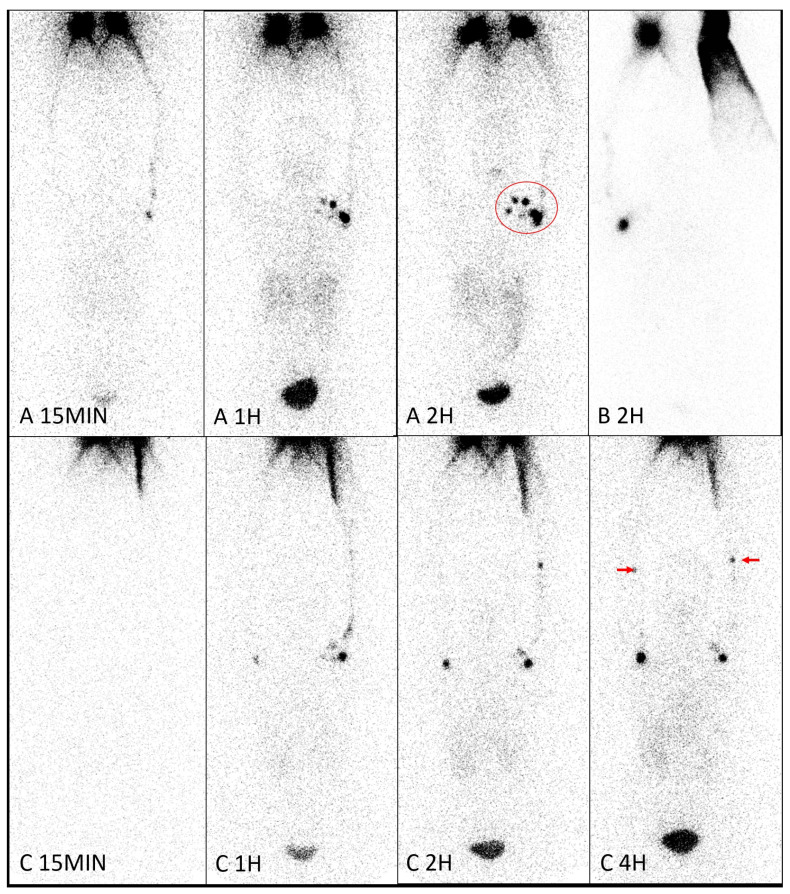
Anterior images of 3 patients (**A**–**C**) demonstrated variation of the normal upper extremity at the left side of patient A and the right side of patients B and C. Up to 5 axillary lymph nodes (large red circle) of patient A’s left upper extremity were counted, with clear visualization and increasing number over time. Forearm nodes were seen in patient C’s upper extremities at 4 h (arrows), one on each side. In patient B, organs, i.e., liver, kidneys, and urinary bladder, are hardly seen even up to 2 h. The findings of upper extremity lymphedema and chronic lymphatic obstruction post-MRM at the right side of patient A and the left side of patient B are as aforementioned in Figure 1’s legend. Although clinical long-term left forearm edema of patient C and some radiotracers accumulate prolongedly in the medial aspect of the left forearm, there is no definite scintigraphy evidence of lymphatic obstruction of patient C’s left upper extremity.

The visual assessment of upper extremity lymph nodes following ^99m^Tc in-house Dextran injection, as detailed in Table 2, indicated the following:

- Axillary lymph node detection rates were 46% within 15 min, rising to 68% between 16 and 30 min, and approximately 86% within the first hour.
- Most identified axillary nodes were solitary, with 13% exhibiting two nodes and 5% showing three or more (Figure 3A).
- Detection in the elbow (Epitrochial/brachial) node was rare, around 4% within an hour, typically involving two or fewer nodes (Figure 3C).
- Delayed imaging up to 4 h further revealed lymph nodes in the axillary region for 10 out of 12 patients initially without detected axillary nodes within the first hour. The remaining two patients did not undergo delayed imaging beyond 2 h.

Table 3 illustrates the detection rates of upper lymphatic pathways and organ activity associated with lymphatic circulation following injection of ^99m^Tc in-house Dextran:

- Upper extremity lymphatic tracts can be detected at approximately 61% within 15 min and up to 92% within 31–45 min.
- Organs such as the liver, kidney, and urinary bladder show detection rates of 54%, 71%, and 93%, respectively, within the first hour (Figure 3B).
- The radiopharmaceutical persists in these organs and lymphatic pathways, with increasing activity observed continuously beyond 50% probability thresholds. Specifically, liver, kidney, and urinary bladder detection thresholds are reached at least 46 min, 31 min, and 16 min, respectively.

### 3.4. Quantitative Analysis

Among the 24 studies conducted, delineation of the ROIs around these areas yielded an initial median count at the axillary nodes of approximately 199.62, which increased to about 922.91 within one hour. In contrast, injection site activity, on the same included hand, started with a median count of approximately 325,377.06 within the first 15 min, gradually decreasing to approximately 237,886.02 counts by the end of 1 h as detailed in Table 4.

From the evaluation of axillary node uptake as a percentage of injection site activity over various time intervals up to 1 h, it was observed that despite the minimal percentage, there is a consistent upward trend in these values, detailed in Table 5. The lymphatic drainage uptake in the axillary nodes shows a consistent increase over time, peaking at approximately 2.5% compared to the activity observed at the injection site within the first hour. Median uptake values at specific time intervals are as follows: within 15 min, 0.05%; 16–30 min, 0.10%; 31–45 min, 0.14%; and reaching a maximum median value of about 0.33% at 1 h. Meanwhile, the median value for radiopharmaceutical clearance in the less-than-15-min interval starts at 2.79%, gradually rising to 9.17% by the end of the first hour.

## 4. Discussion

Lymphoscintigraphy is a nuclear medicine imaging technique employed to assess the lymphatic system, used for sentinel lymph node localization and lymphedema evaluation of upper or lower extremities [30,31,32,33,34,35], utilizing radionuclide-labeled colloidal particles [36,37]. Presently favored and commercially available agents include ^99m^Tc-filtered sulfur colloid, characterized by particle sizes ranging from 10 to 50 nanometers [31]. In contrast, ^99m^Tc-albumin nanocolloid, featuring particles smaller than 80 nanometers, often encounters challenges during labeling [30]. ^99m^Tc-human serum albumin (HSA) and ^99m^Tc-dextran exhibit rapid movement with particle sizes less than 4 nanometers. As previously mentioned, the local Dextran kit is different. Therefore, we have ^99m^Tc in-house Dextran with colloidal particle sizes of approximately 15-40 nanometers. Various factors besides colloidal size influence distribution within the lymphatic system. These may include flow dynamics between lymphatic vessel walls and nodes, affected by vascular and interstitial characteristics, as well as muscular activities [38,39,40]. The advantages and disadvantages of various techniques have been thoroughly reviewed [9]. For characterization or follow-up purposes, the techniques employed should be consistent.

This descriptive retrospective study aims to analyze the characteristics and dynamics of intradermally injected ^99m^Tc in-house Dextran distribution through the lymphatic system, visualized from raised hands to the body via lymphoscintigraphy. The study population primarily consisted of breast cancer patients with unilateral arm lymphedema following surgery, with the contralateral arm serving as the studied population of normal upper extremity. The cohort was predominantly from late middle-aged individuals to early seniors, with an average BMI indicating an overweight status. Both upper extremities were assessed qualitatively and quantitatively in a balanced manner.

Evaluation of upper extremity lymph node visualization following injection of the ^99m^Tc in-house Dextran radiopharmaceutical via lymphoscintigraphy revealed that axillary lymph nodes were observed in approximately 46% of cases within less than 15 min, and an additional 40% between 16 min and 1 h, totaling 86%. These findings closely mirrored studies by Rezende et al. (2011) [41] and de Oliveira et al. (2016) [42] using ^99m^Tc-dextran, which reported node visualization rates of approximately 44% and 39.42% within the first 10 min, totaling 88% and 77% at 1 h, respectively. In contrast, studies using ^99m^Tc-nanocolloid by Rossi et al. (2016) [43] indicated slower movement with lymphatic nodes observed in normal arms at approximately 20% within the first 20 min, totaling 50% at 1 h, attributable to larger particle sizes.

Axillary lymph node visualization was achieved in nearly all patients within this study, although in some cases, visibility extended beyond the first hour, with delays of up to 4 h noted. However, two patients did not reveal upper extremity lymph nodes within the initial 2-h imaging period. Nonetheless, visualization of lymphatic drainage pathways and organ uptake continued, as depicted in Figure 1.

A single axillary node was most frequently visualized, with the highest count observed being five nodes (Figure 3A). Imaging consistently showed a high likelihood of finding one node (70%), two nodes (23%), and three or more nodes (5%) within 4 h post-injection. This contrasts with findings from Devoogdt et al. (2014) [44], utilizing ^99m^Tc-HSA, which reported a higher likelihood (25%) of visualizing four nodes and an 18% probability of detecting a single node, attributed to smaller particle sizes and subcutaneous injection.

Visual assessments of the upper extremity lymphatic tract indicated up to 92% visibility within 30 min post-injection. Further tracer accumulation in organs associated with lymphatic circulation is evident in the urinary bladder, kidney, and liver, respectively. This suggests rapid renal excretion of the radiopharmaceutical, with transient accumulation in the renal parenchyma. Activity persisted in lymphatic pathways and organs within the first hour, gradually increasing, with visibility exceeding 50% in the liver, kidneys, and urinary bladder at least 46 min, 31 min, and 16 min post-injection, respectively, in line with findings by de Oliveira et al. (2016) [42]. The lymphatic pathway and organs are less visible in larger radiocolloid use of ^99m^Tc-phytate [45].

Quantitative analysis of axillary lymph node counts indicated initial median counts of approximately 199.62 within the first 15 min, progressively increasing over time. By 1 h, counts reached a maximum median of 922.91. The median percentage of axillary lymph node uptake (%ANU) at 31–45 min approximated 0.14%, comparable to findings by Devoogdt et al. (2014) [44] utilizing ^99m^Tc-HSA, where median values were around 0.1% at 45 min. Axillary node uptake percentages remain around 1% with other radiocolloids [29,45]; however, in cases of lymphedema, the affected arm shows lower uptake percentages compared to the normal arm when using the same radiocolloid.

The median percentage of the ^99m^Tc in-house Dextran clearance from the intradermally injected hand was estimated at approximately 9% within the first hour. Conversely, Devoogdt et al. (2014) [44], using ^99m^Tc-HSA, reported a median radiopharmaceutical clearance of 5-7% at 2 h post subcutaneous injection. Similarly, the radiotracer clearance percentage is lower in the lymphedema-affected arm compared to the normal arm [29].

The limitations of this study include its retrospective design, which has potential inconsistencies in documentation. Imaging predominantly occurred within the first hour post radiocolloid injection, possibly limiting observations beyond this period. Sample size may also have affected the study’s ability to fully assess qualitative and quantitative aspects. Furthermore, findings related to organ activity in the liver, kidneys, and urinary bladder primarily pertain to breast cancer patients who had undergone surgery unilaterally, potentially limiting generalizability to the broader population.

The selection of radiocolloid types, injection sites, and imaging protocols has been discussed [46]. These decisions typically revolve around cost-effectiveness considerations and radiocolloids’ local availability [28]. These factors are crucial in determining the feasibility and practicality of lymphoscintigraphy procedures in clinical practice.

## 5. Conclusions

This study characterizes normal upper extremity lymphatic drainage using ^99m^Tc in-house Dextran, offering insights into its clinical application.

## Figures and Tables

**Table 1:** Population characteristics.

Normal Upper Extremity	Qualitative Analysis (*n*= 84)	Quantitative Analysis (*n* = 24)
Age (years), mean ± SD	59.96 ± 12.15	62.87 ± 11.92
Weight (kg), mean ± SD	64.36 ± 12.20	65.41 ± 10.66
Height (cm), mean ± SD	154.26 ± 6.00	154.26 ± 6.39
BMI (kg/m^2^), mean ± SD	26.92 ± 4.64	27.20 ± 4.14
Gender - Female, *n* (%)	81 (96%)	24 (100%)
Normal arm - Right, *n* (%)	42 (50%)	11 (45%)
Underlying breast cancer, *n* (%)	77 (92%)	23 (96%)

**Table 2:** Probability of detecting upper extremity lymph nodes from lymphoscintigraphy images (*n* = 84).

Time Interval (min)	Nodal Number	Regions	
		Axillary, *n* (%)	Epitrochial/brachial, *n* (%)
≤15	0	45 (54%)	83 (99%)
	1	33 (39%)	1 (1%)
	2	4 (5%)	-
	≥3	2 (2%)	-
16–30	0	27 (32%)	82 (98%)
	1	45 (53%)	1 (1%)
	2	9 (11%)	1 (1%)
	≥3	3 (4%)	-
31–45	0	16 (19%)	81 (96%)
	1	54 (64%)	2 (3%)
	2	10 (12%)	1 (1%)
	≥3	4 (5%)	-
46–60	0	12 (14%)	81 (96%)
	1	57 (68%)	2 (3%)
	2	11 (13%)	1 (1%)
	≥3	4 (5%)	-
240 (Up to 4 h)	0	2 * (2%)	81 (96%)
	1	59 (70%)	2 (3%)
	2	19 (23%)	1 (1%)
	≥3	4 (5%)	-

* no 4-h-delayed imaging.

**Table 3:** Probability of detecting lymphatic pathways and organs associated with lymphatic circulation.

Visualization	Time Interval (min)			
	≤15	16–30	31–45	46–60
Upper extremity lymphatic tract activity, *n* (%)	51 (61%)	70 (83%)	77 (92%)	77 (92%)
Liver activity, *n* (%)	8 (10%)	17 (20%)	23 (27%)	45 (54%)
Kidney activity, *n* (%)	10 (12%)	31 (37%)	45 (54%)	60 (71%)
Urinary bladder activity, *n* (%)	23 (27%)	65 (77%)	73 (87%)	78 (93%)

**Table 4:** Quantitative evaluation of axillary lymph node and injection site activity (*n* = 24).

Time Interval (min)	Study of Visualized Lymph Node, *n* (%)	Total Count [Median (Range)]	
		Axillary Lymph Node	Injection Site
≤15	14 (58%)	199.62 (2377.43)	325,377.06 (446,079.03)
16–30	16 (67%)	468.84 (4090.21)	302,932.48 (467,057.55)
31–45	18 (75%)	508.01 (5794.04)	272,720.90 (457,650.09)
46–60	20 (83%)	922.91 (6655.25)	237,886.02 (398,128.31)

**Table 5:** Uptake of lymphatic drainage pathway to axillary node and clearance at injection site.

Time Interval (min)	% Axillary Lymph Node Uptake (ANU) [Median (Range)]	% Radiopharmaceutical Clearance (RC) [Median (Range)]
≤15	0.05 (1.46)	2.79 (4.19)
16–30	0.10 (1.56)	3.18 (56.96)
31–45	0.14 (2.06)	7.02 (83.71)
46–60	0.33 (2.40)	9.17 (50.03)

## Data Availability

The data presented in this study are available on request from the corresponding author.
